# Advances in GNSS Positioning and GNSS Remote Sensing

**DOI:** 10.3390/s24041200

**Published:** 2024-02-12

**Authors:** Yury V. Yasyukevich, Baocheng Zhang, Venkata Ratnam Devanaboyina

**Affiliations:** 1 Institute of Solar-Terrestrial Physics SB RAS, Irkutsk 664033, Russia; 2 Innovation Academy for Precision Measurement Science and Technology, Chinese Academy of Sciences, Wuhan 430077, China; b.zhang@whigg.ac.cn; 3 Department of Electronics and Communication Engineering, Koneru Lakshmaiah Education Foundation, Green Fileds, Vaddeswaram 522302, India; ratnam2002v@gmail.com

Scientists and engineers use data utilize global navigation satellite systems (GNSSs) for a multitude of tasks: autonomous navigation, transport monitoring, construction, GNSS reflectometry, GNSS ionosphere monitoring, etc. To improve the precision of GNSSs, many devices combine different sensors and GNSS receivers, scientists have found ways to enhance GNSS receivers (and satellites), and mathematicians have improved navigation solutions. Combining different navigation systems (GPS, GLONASS, Galileo, BeiDou) has also facilitated the achievement of precise positioning and remote sensing.

GNSSs offer various PNT applications in the domains of aviation, maritime, and land. Engineers have attempted to implement autonomous GNSSs in the mass market [1]. Autonomous vehicles (especially in the urban environment) have expanded the need for precise navigation. The existing real-time high-precision GNSS positioning services available to public users continue to face challenges. The main challenges are as follows:

1. *Low cost*. Precise geodetic GNSS receivers are expensive. The mass market requires low-cost receivers such as u-blox receivers and smartphone-level GNSS chips [2,3]. It is crucial that performance and application tests are conducted for these low-cost GNSS devices for public GNSS services.

2. *High accuracy*. Due to cost issues, mass market receivers mainly employ noisy code observations, which result in large range errors [1]. Meter-level positioning based on single-frequency code observations cannot satisfy the mass market. It is therefore important that research achieves centimetre-level positioning accuracy based on the precise phase observations obtained from low-cost receivers [4].

3. *Reliability*. Some GNSS applications (such as autonomous vehicles) require high reliability (including accuracy, integrity, and availability), so they crucially depend on the monitoring of integrity [5]. Determining how to ensure stable and continuous precise GNSS positioning for the general public is worth study. For weak GNSS legacy signals that lack authentication, Radio Frequency Interference (RFI) is another reliability problem; this comprises:

- Natural threats such as space weather events including geomagnetic and ionospheric storms, solar flares, ionospheric scintillations [6]. The upcoming solar cycle 25 maximum could cause adverse space weather events that degrade the GNSS signals (the GNSS RFI threats have increased rapidly since 2018: EASA, the EU Aviation Safety Agency, and the European Common Repository reported 4689 GNSS Events in 2022 and 4147 GNSS Events in the first half of 2023 due to tense international situations [7]).
- Intentional threats (Jamming and spoofing).
- Unintentional interferences (LTE700 band).

Research addressing the three aspects mentioned above will greatly promote the application of real-time high-precision GNSS positioning in the mass market and advance autonomous driving and urban digitization. This Special Issue, entitled “*Advances in GNSS Positioning and GNSS Remote Sensing”* in *Sensors* (https://www.mdpi.com/journal/sensors/special_issues/AGPGRS, (accessed on 2 February 2024)), attracted 10 articles [8,9,10,11,12,13,14,15,16,17] on new GNSS techniques for ionospheric studies, the impact of space weather on GNSS and methods for precise navigation.

He et al. [8] suggested the use of an index to estimate the effects of space weather on BeiDou; this is entitled the Percentage of Affected Satellites (PAS). The index depends on the relative difference between observed satellites and those *P_SLGS_* that are theoretically predicted, the relative number of observations with a loss-of-lock indicator alert *P_LLI_*, and the relative number of so-called total electron content (TEC) slips (this term is used according to [18]), i.e., when the geometry-free ionospheric combination exceeds a limit, *P_GF_*. The index is normalized by the number of (affected) stations *N_S_*.

(1) $$PAS=\frac{\sum_{n=1}^{N_{S}}P_{SLGS}+\sum_{n=1}^{N_{S}}P_{LLI}+\sum_{n=1}^{N_{S}}P_{GF}}{N_{S}}$$

Here, we slightly changed the equation, excluding coefficients k from the equation in the initial article.

The suggested index expands the approaches introduced by E. Afraimovich and E. Astafyeva [18,19]. *ROTI* and *PAS* were correlated at ~0.9% [8]; however, *PAS* was thought to be superior for disturbed time because, due to the loss of lock (even for a single epoch within 5 min), we could not calculate *ROTI*. The Pearson correlation coefficients for *PAS* with *Kp* or *Dst* were ~0.85. The PPP errors for low (<30%) and high (>30%) PASes, which indicated a huge increase in the positioning error during magnetic storms. While this proves previous results regarding geomagnetic storms [6], it is impossible to make a prediction without large statistics. We feel that this index could be an appropriate indicator and suggest that a model for PPP quality alerts based on *PAS* is made. A shortcoming of the research is the small number of statistics considered; the authors considered only one magnetic storm (12 May 2021), but the results are promising and we anticipate the publication of additional papers demonstrating different aspects of the suggested index.

Because most GNSS users still apply single-frequency equipment [20], it is vital that the ionospheric models more suitable for ionospheric range correction are determined. Rovira-Garcia et al. [21] showed that the positioning domain could be a reliable factor. In the current Special Issue, an article by an international team from Russia, China and Italy [9] compares nine ionosphere models, namely Klobuchar, NeQuickG, BDGIM, GLONASS, IRI-2016, IRI-2012, IRI-Plas, NeQuick2, GEMTEC, from two perspectives: (1) how accurately the models can calculate TEC, and (2) how precisely the single-frequency receivers can calculate the position. The article contains a brief description of the models and various information, including the statistical distributions, precision dependencies from time, Kp, F10.7, local time, and latitude. The authors align the models, with regard to the positioning error, against the noncorrected solution from the mean absolute error and root-mean-square error as GIM IGSG, BDGIM, NeQuick2, GEMTEC, NeQuickG and IRI-2016, Klobuchar, IRI-2012, IRI-Plas, GLONASS, and in the TEC domain as GEMTEC, BDGIM, NeQuick2, IRI-2012, NeQuickG, IRI-2016, Klobuchar, GLONASS. However, the peculiarities of error distribution could have resulted in differences in the mean absolute percentage error (see Figure 1). The obtained results revealed that (1) the quality should be estimated for the region in which one is going to use a model, that (2) we should use several parameters to estimate the quality of a model, and that (3) we still need a procedure to compare different ionospheric models.

Two articles [10,11] address the global ionospheric weather and global electron content (*GEC*, suggested by E. Afraimovich [22]). We would like to add that the *GEC* is routinely calculated and is freely available via SIMuRG (https://simurg.space/rec, accessed date: 1 February 2024) [23].

An international team from Russia, Spain, and Poland investigated the SpaceX magnetic storms [10], namely, the storms that occurred when SpaceX launched its Starlink satellites (on 3 February 2022 and 7 July 2022). For these case studies, the IZMIRAN and CAS prediction products seemed to perform a little better than those produced by CODE and Beijing University of Aeronautics and Astronautics. The authors suggest that the GEC is a good indicator of space weather, but insist that the community requires a significant improvement in the forecast of global ionospheric maps (used for GEC calculation). The article shows that a reliable ionosphere forecast is not currently available. We believe that by combining the approaches proposed in [9,10], the GNSS community could obtain more reliable estimates and overcome the related problems.

In their study, Aroca-Farrerons et al. suggest the utilization of the GEC spectrum as an indicator of space weather [11]. Compared with the papers mentioned [22,23], Aroca-Farrerons et al. [11] used short-term spectra. The 15-minute UQRG GIM enhanced the suggested approach. The authors assessed 34 magnetic storms occurring in 2000–2020 to find that >50% of the spectrograms of the *GEC* and *Kp* are correlated and that <21% of spectrograms are not correlated. Their most important finding was that the *GEC* and *Kp* spectrums correlated better than their time series. This indicates that the GEC spectrum could be a better indicator of space weather than the GEC itself.

In their study, Bronk et al. [12] suggest that a risk assessment analysis of GNSS threats (intentional and unintentional) be performed for the upcoming Galileo Public Regulated Service (PRS) in Poland. The paper highlights how the amateur services within the 1240–1300 MHz band could interfere with the Galileo E6 (central frequency—1278.75 MHz). Poland contributes to two international research collaborative PRS projects—(1) the PRS Pilot Project for Demonstration (3PfD) and the (2) GNSS Interference Monitoring and Mitigation for End Users–PRS (GIMME PRS)—and has built a waveform database containing the potential PRS signal interferers. A Galileo PRS Threats Detection system is proposed, and the GNSS Jamming Test results in a Controlled Laboratory Environment with various GNSS smartphones and chipsets under various interference scenarios are presented. Bronk et al. [12] found that the resistance observed to various jamming signals depends on the manufacturer of the GNSS receiver, and suggestive measures are discussed regarding the importance of the national systems employed in GNSS threat detection.

Krietemeyer et al. [13] developed a web-based tool for GNSS Antenna phase centre offset (PCO) calibration for low-cost GNSS receivers and antennas. The PCO calibration procedure employed an elevation-based residual averaging method and a short baseline. In order to conduct the antenna calibration tests, the authors used different GNSS antennas to evaluate the stability of the positioning and offsets. The results revealed that a 1° binning width represents a good trade-off between effectiveness and smoothing when compared to other binning widths. The online tool accepts GNSS RINEX [24,25] files and is compatible with IGS ANTEX standard tropospheric, atmospheric, and crustal deformation monitoring systems.

In their study, Islam et al. [14] suggest the utilization of a jamming detector with multi-frequency and multi-constellation software that was able to define the GNSS receiver for Maritime Navigation in the Gulf of Finland. GPS-L5-only, Galileo-E5a-only, and Galileo-E5b-only signals and their multi-GNSS combination positioning results revealed that the Galileo-E5 and E5b signals performed better than other frequency bands in a maritime operational environment. By utilizing different signals, it is easier to combat jamming, especially if it does not cover all the GNSS signals. This paper suggests that a jamming impact analysis of GPS L5 signals is performed with full constellation and E5 full-band AltBoC signals.

Hamza et al. [15] provide a comprehensive examination of the utilization of low-cost GNSS receivers in positioning applications. Their research focuses on a comparison of these consumer-grade GNSS devices with high-quality geodetic GNSS devices, considering the carrier-to-noise ratio (C/N0), multipath errors, and their overall positioning accuracy in different environments. The results revealed that low-cost GNSS instruments exhibit a promising performance, achieving a horizontal accuracy below 10 mm in urban areas for a sizeable portion of the sessions. These findings are crucial for expanding the application of GNSS technology in public GNSS services, offering a low-cost GNSS service option with an impressive performance.

Swaminathan et al. [16] thoroughly examined the techniques utilized to augment the GNSS position within the challenging urban scenario under three modes: differential GNSS, Real-time Kinematic (RTK), and Real-time eXtended (RTX). Using the Applanix POS-LV 220 navigation system and high-definition maps for validation, they studied how the receiver navigates through diverse scenarios, including uneven terrain, tall buildings, varying road widths, and tunnels. They found that the RTX method overcame RTK, displaying centimeter precision in urban environments. The study found that RTX is a reliable and precise position augmentation technique for the urban environment and can be employed to advance GNSS-based autonomous vehicle applications for the mass market.

Kim et al. [17] focuses on a pivotal element of the Centimeter-Level Augmentation System (CLAS) in the Quasi-Zenith Satellite System (QZSS): the formulation of a protection level equation for PPP-RTK methods. Unlike other GNSS augmentation systems, the proposed equation integrates considerations for correct integer ambiguity fixes in GNSS carrier-phase measurements and CLAS correction quality messages. The research utilizes GNSS Earth Observation Network (GEONET) stations in Japan and CLAS broadcast messages to experimentally compare the computed protection levels with the position errors. The results, which spanned a 7-day dataset, demonstrated that the protection levels derived from the proposed equations consistently exceeded the position errors. The RMS errors of the CLAS Virtual Reference Station-Real Time Kinematic (VRS-RTK) positions were 4.6 cm and 14 cm in the horizontal and vertical directions, respectively. This study significantly advances the development of integrity monitoring solutions, establishing a foundation for reliability in GNSS positioning services for the mass market.

The applied tasks require further scientific developments in this field. We hope that the articles published in this Special Issue will help to solve these scientific and applied problems.

## Figures and Tables

**Figure 1:**
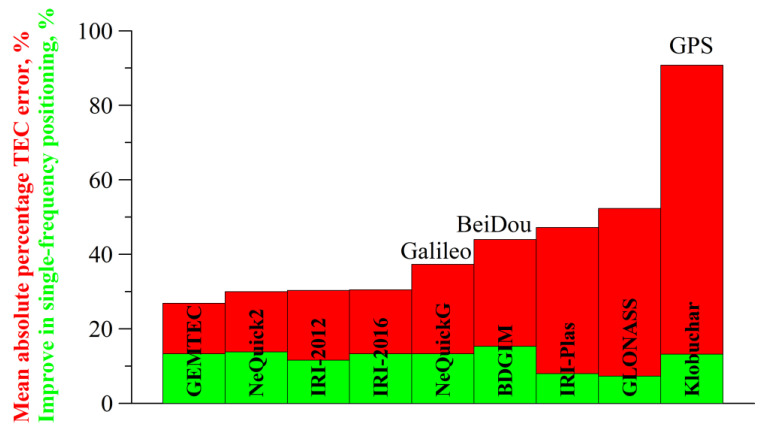
The quality of nine ionospheric models (aggregating the data from Table 2 in [9]). The single-frequency positioning accuracy improvements are shown in green, the mean absolute percentage TEC errors are shown in red.
